# Transient-Absorption Pump-Probe Spectra as Information-Rich Observables: Case Study of Fulvene

**DOI:** 10.3390/molecules30071439

**Published:** 2025-03-24

**Authors:** Zhaofa Li, Jiawei Peng, Yifei Zhu, Chao Xu, Maxim F. Gelin, Feng Long Gu, Zhenggang Lan

**Affiliations:** 1 School of Chemistry, South China Normal University, Guangzhou 510006, China; precalcolus@outlook.com; 2 MOE Key Laboratory of Environmental Theoretical Chemistry, SCNU Environmental Research Institute, Guangdong Provincial Key Laboratory of Chemical Pollution and Environmental Safety, School of Environment, South China Normal University, Guangzhou 510006, China; pengjw@aliyun.com (J.P.); zhuyifei.phil@gmail.com (Y.Z.); chaoxu@m.scnu.edu.cn (C.X.); 3 School of Science, Hangzhou Dianzi University, Hangzhou 310018, China

**Keywords:** conical intersections, transient absorption pump-probe spectroscopy, symmetrical quasiclassical/Meyer–Miller–Stock–Thoss, doorway window approximation

## Abstract

Conical intersections (CIs) are the most efficient channels of photodeactivation and energy transfer, while femtosecond spectroscopy is the main experimental tool delivering information on molecular CI-driven photoinduced processes. In this work, we undertake a comprehensive ab initio investigation of the CI-mediated internal conversion in fulvene by simulating evolutions of electronic populations, bond lengths and angles, and time-resolved transient absorption (TA) pump-probe (PP) spectra. TA PP spectra are evaluated on the fly by combining the symmetrical quasiclassical/Meyer–Miller–Stock–Thoss (SQC/MMST) dynamics and the doorway-window representation of spectroscopic signals. We show that the simulated time-resolved TA PP spectra reveal not only the population dynamics but also the key nuclear motions as well as mode–mode couplings. We also demonstrate that TA PP signals are not only experimental observables: They can also be considered as information-rich purely theoretical observables, which deliver more information on the CI-driven dynamics than conventional electronic populations. This information can be extracted by the appropriate theoretical analyses of time-resolved TA PP signals.

## 1. Introduction

Nonadiabatic processes taking place near crossing areas of potential energy surfaces (PESs) are ubiquitous in photochemistry and photobiology [1,2,3,4,5,6]. When two electronic states have the same multiplicity, conical intersections (CIs), i.e., degeneracy areas between adjacent adiabatic PESs, facilitate radiationless transitions [7] and population transfer [8]. CI-driven nonadiabatic processes occur, usually, on the femtosecond timescale [3,8]. However, the theoretical treatment of the nonadiabatic dynamics is not a trivial task because of the strongly coupled nuclear-electronic motions. In this case, the well-known Born–Oppenheimer approximation breaks down, and the effective dynamics approaches should be developed to simulate the nonadiabatic transitions. It is thus important but challenging to determine microscopic mechanisms and molecular motions governing the CI-driven wavepacket dynamics.

Nowadays, femtosecond spectroscopy is the main experimental source of information on ultrafast photoinduced processes [9,10,11,12,13,14,15,16,17], and transient-absorption (TA) pump-probe (PP) spectroscopy is one of the most widespread techniques [18,19,20,21,22,23,24]. In TA PP experiments, the pump pulse interacts with molecular systems, creates the wavepacket in the molecular excited states, and initializes the further dynamics. After a certain time delay, the probe pulse interacts with the system under study, producing the signals. By adjusting the time delay, the PP experiments can be employed to detect the dynamical/reaction processes. Note that the TA PP signal is, in fact, a difference of two signals, detected with and without pump pulse [16]. More specifically, the TA PP spectra consist of three contributions, i.e., the ground state bleach (GSB), the stimulated emission (SE), and the excited state absorption (ESA). The SE and ESA signals monitor the excited-state molecular motions by projecting the nuclear wavepacket in the lower-lying excited states to the ground state (SE) and the higher excited states (ESA). The ground state bleach (GSB) has two components: the cold component reflects the ground-state wavepacket motion, and the hot component contains information on molecular motions after the internal conversion to the ground state [25]. Overall, TA PP spectroscopy delivers information on the wavepacket dynamics in the (coupled) ground and lower-lying excited electronic states. The information is encoded into, e.g., integral TA PP spectra $I_{int}\left(\tau ,\omega_{pr}\right)$ [26], which are monitored as a function of the time delay between the pump and probe pulses (so-called population time τ) and probe frequency $\omega_{pr}$, but depend implicitly on the excitation frequency $\omega_{pu}$, durations of the PP pulses, and parameters describing the molecular system under study. However, it is impossible to comprehensively characterize photoinduced dynamics at CIs by using TA PP spectra alone: theoretical support is essential for their interpretation. Thus, simulation of time-resolved spectra is important because it helps us to extract the essential information on nonadiabatic dynamics near CIs [27,28,29,30,31]. Consequently, considerable efforts were devoted to simulate nonlinear time-resolved signals within the framework of quantum dynamics [32,33], hierarchical equations of motion (HEOM) [34], mixed quantum-classical Liouville equations [35,36], surface hopping [37,38,39,40], and Ehrenfest dynamics [41]. These efforts were aimed to build a more straightforward connection between nonadiabatic dynamics driven by CIs and time-resolved signals [26,42,43,44,45,46,47,48,49]. For small or model systems, it is possible to use many of the methods to treat the nonadiabatic dynamics with different accuracies. However, in high-dimensional systems with strong anharmonicities, significant couplings between different degrees of freedom, and arbitrary molecular motions, many of the above methods become unfeasible. In this situation, the on-the-fly simulations become attractive.

With the advancement of computational facilities and theoretical algorithms, efforts of many groups have been shifted to the construction of on-the-fly protocols for simulations of spectroscopic signals for realistic molecular systems [46,49,50,51,52,53,54,55,56,57,58]. These efforts open the possibility to microscopically understand ultrafast time-resolved spectra of polyatomic systems and to obtain insight into nonadiabatic dynamics involving large-amplitude molecular motions.

Usually, the on-the-fly simulations are conducted within the trajectory-based dynamics formulism. Within this framework, arbitrary nuclear motions are described by classical mechanics, and the nonadiabatic effects are treated with different approximate methods. Therefore, this approach is very popular in the exploration of nonadiabatic processes of polyatomic systems due to its balance of efficiency and accuracy [44]. Among them, the quasiclassical or semiclassical dynamics methods based on the mapping Hamiltonian [59,60,61] received considerable attentions. Within the mapping framework, a quantum system with several discrete levels is transformed into a system with coupled continuous degrees. Then, different quasiclassical or semiclassical dynamics approaches have been developed. Different versions of the mapping approach were implemented to study nonadiabatic dynamics at CIs in recent decades [62,63,64,65,66,67,68,69,70,71,72,73]. Within the mapping framework, different theoretical approaches were proposed to simulate different types of nonlinear spectroscopic signals [74,75,76,77,78,79,80,81,82,83,84,85,86,87,88,89,90]. It is to be noted that the direct simulation of spectroscopic third-order response functions by the mapping approaches is quite tedious even for model systems [47]. On the other hand, the direct inclusion of laser fields at different time delays in the propagation is also computationally costly [91,92,93,94]. In simple words, more rigorous methods generally have to face larger computational costs. It is thus worthwhile to invoke some alternative, practical ideas for the simulation of time-resolved spectra [49].

The doorway-window (DW) approximation [95,96,97,98,99] accounting for finite duration and arbitrary spectral shapes of the pump and probe pulses provides a practical approach for the evaluation of spectroscopic signals. In the DW approximation, the pump and probe pulses are well temporally separated and short on the nuclear dynamics timescale. This approximation is satisfied in many femtosecond spectroscopy experiments. In this DW approximation, calculations of the spectroscopic signals are performed in three steps: construction of the doorway operator, construction of the window operator, and field-free system propagation. Here, the doorway operators describe the interaction between the pump pulse and the molecular system, the system Hamiltonian governs the evolution of the system after the initial preparation, and the window operators describe the interaction between the probe pulse and the molecular system. This approximation can conveniently be interfaced with any ab initio trajectory method: As the input, the DW protocol requires exclusively electronic energies and transition dipole moments (TDMs) along trajectories. So far, the ab initio DW methodology has been combined with two commonly used simulation techniques: surface hopping (Tully’s fewest switches [100,101,102], Landau–Zener [25,103], and machine-learning-enhanced Landau–Zener [104]) and Ehrenfest [105].

Recently, a combination of symmetrical quasiclassical/Meyer-Miller-Stock-Thoss (SQC/MMST) dynamics with the DW approximation has been proposed for the simulation of TA PP signals [106]. Clearly, the SQC/MMST approach permits one to calculate nonadiabatic wavepacket dynamics with (usually) sufficient accuracy and at affordable computational costs [85,107,108,109,110,111,112]. In some popular trajectory dynamics methods like surface hopping and Ehrenfest dynamics, nuclear and electronic motions are described, respectively, classically, and quantum mechanically. In the mapping approaches, however, both nuclear and electronic motions are described classically. Furthermore, the mapping approach may have advantages over the surface hopping and Ehrenfest counterparts in reproducing electronic coherences without the ad hoc inclusion of decoherence corrections, which is essential for the simulation of TA PP and other nonlinear spectroscopic signals. This can become essential for the adequate simulation of TA PP and other nonlinear spectroscopic signals. In addition, the mapping approach can be reformulated in the adiabatic representation [113] and conveniently used in the on-the-fly simulations to describe realistic molecular systems [113,114,115,116].

In this work, we simulated TA PP signals by ab initio methods through interfacing the DW approximation and the SQC/MMST dynamics (DW-SQC/MMST). The work has both theoretical and methodological dimensions. On the methodological side, we further extend the application base of the on-the-fly DW simulation protocol [25].

On the theoretical side, we explore the possibility of abstracting the key information about the nuclear motions involved in the CI-driven nonadiabatic dynamics by combined on-the-fly simulation of the traditional dynamical observables (electronic populations, bond lengths, and bond angles) and TA PP spectra.

The fulvene molecule was chosen as a typical CI-driven molecular system, which is frequently used as a real-world representative of “Tully’s third model” [117]. The photoinduced dynamics in fulvene are fairly well understood at the ab initio level [118,119,120,121,122,123,124,125,126,127,127]. This allows us to concentrate on squeezing additional information from the TA PP signals. It is essential that surface hopping methods do not give a very precise description of fulvene’s population dynamics [118,119,120,121], while the SQC/MMST method with a triangular window function provides results that are consistent with ab initio multiple spawning (AIMS) data [121]. This lends support to the choosing of the SQC/MMST method for simulations of TA PP signals of fulvene. Our results show that the TA PP signals directly reflect the key molecular motions in the nonadiabatic dynamics of fulvene. This work thus provides useful ideas on how to connect ultrafast spectroscopic signals with the essential features in nonadiabatic dynamics.

## 2. Theoretical Methods and Computational Details

### 2.1. SQC/MMST Approach

Mapping models, in which an *F*-level quantum system is mapped to a system with *F* coupled continuous degrees of freedom (DoFs), provide a practical way for the construction of classical-like dynamics approaches for the simulation of quantum evolutions of a realistic large system [65,68]. In the MMST model [59,60,61], a set of discrete quantum states is mapped to a group of coupled classical oscillators. With the SQC protocol, the initial sampling and the final assignment of the electronic DoFs are performed through the symmetrical triangle window function [128,129]. More rigorous derivations can be found in the previous works [60,110,130,131]. By combining the MMST mapping model with the SQC protocols, the SQC/MMST approach allows us to simulate the nonadiabatic dynamics [107,108] with acceptable accuracy and affordable computational costs [109,111,112]. Since the SQC/MMST method is easy to reformulate in the adiabatic representation [113], it can be conveniently combined with the ab initio quantum chemistry software to describe the nonadiabatic dynamics in realistic molecular systems [114,115,116] by using the trajectory-adjusted zero-point energy (ZPE) correction $\gamma_{i}$ [113,132].

For the molecular system, the Hamiltonian $\hat{H}$ of *F* states can be formulated in

(1) $$\begin{array}{c} \hat{H}=\sum_{ij}{\hat{H}}_{ij}|\phi_{i}\rangle \langle \phi_{j}|, \end{array}$$

where $\phi_{i}$ and $\phi_{j}$ represent electronic states of the system, diagonal ${\hat{H}}_{ii}$ is the energy operator, off-diagonal elements ${\hat{H}}_{ij}$ denote the interstate coupling operators. The idea of mapping approaches is to map the discrete representation to the continuous representation. In the MMST model, an *F*-state system is mapped to *F* coupled harmonic oscillators. The creation operator ${\hat{a}}_{i}^{†}$ and annihilation operator ${\hat{a}}_{j}$ are employed to construct the mapping

(2) $$\begin{array}{cc} \; & |\phi_{i}\rangle \langle \phi_{j}|\mapsto {\hat{a}}_{i}^{†}{\hat{a}}_{j},\\ \; & |\phi_{i}\rangle \mapsto |0_{1}\ldots 1_{i}\ldots 0_{k}\ldots 0_{F}\rangle , \end{array}$$

where $1_{i}$ means that the occupation number of *i*-state is 1, and $0_{k}$ means that the occupation number of *k* state is 0. The creation and annihilation operators satisfy the relation $\left[{\hat{a}}_{j},{\hat{a}}_{i}^{†}\right]=\delta_{ji}$. The coordinate and momentum operators are defined as ${\hat{x}}_{i}=\left({\hat{a}}_{i}^{†}+{\hat{a}}_{i}\right)/\sqrt{2}$ and ${\hat{p}}_{i}=i\left({\hat{a}}_{i}^{†}-{\hat{a}}_{i}\right)/\sqrt{2}$, respectively. Hence, $\hat{H}$ in the diabatic representation becomes

(3) $$\begin{array}{cc} \hat{H}= & \sum_{i}\left[\frac{1}{2}\left({\hat{x}}_{i}^{2}+{\hat{p}}_{i}^{2}\right)-\frac{1}{2}\right]{\hat{H}}_{ii}+\frac{1}{2}\sum_{i\neq j}\left({\hat{x}}_{i}{\hat{x}}_{j}+{\hat{p}}_{i}{\hat{p}}_{j}\right){\hat{H}}_{ij}, \end{array}$$

where $\frac{1}{2}$ is quantum zero point energy (ZPE) and $\hat{H}$ can be viewed as a system of coupled quantum oscillators. By substituting quantum operators with their classical counterparts, we obtain the mapping Hamiltonian *H* that describes coupled classical oscillators. The energy of a classical oscillator, however, can be lower than its ZPE. In this case, unphysical phenomena arise, such as spurious conformation changes caused by ZPE energy transfer from stiff modes to soft modes. The most efficient and a simple strategy for this problem is to reduce the full ZPE to a smaller value by introducing the ZPE correction γ [133]. Therefore, the mapping Hamiltonian *H* can be written as

(4) $$\begin{array}{c} H=\sum_{i}\left[\frac{1}{2}\left(x_{i}^{2}+p_{i}^{2}\right)-\gamma \right]H_{ii}\left(R,P\right)+\frac{1}{2}\sum_{i\neq j}\left(x_{i}x_{j}+p_{i}p_{j}\right)H_{ij}\left(R,P\right), \end{array}$$

where R denotes nuclear coordinates, P denotes nuclear momenta, $x_{i}$ is a mapping electronic coordinate, $p_{i}$ is a mapping electronic momentum, and $H_{ii}$ and $H_{ij}$ are classical analogues of quantum ${\hat{H}}_{ii}$ and ${\hat{H}}_{ij}$, respectively. We can perform initial sampling and launch classical dynamics along trajectories from the mapping Hamiltonian *H*, which enables the nonadiabatic dynamics simulation at the quasiclassical level. By canonical transformation, Equation (4) can be changed to the adiabatic representation [113].

The classical MMST Hamiltonian in the adiabatic representation reads,

(5) $$\begin{array}{cc} H & =\frac{1}{2M}P_{kin}^{2}+\sum_{i}^{F}\left[\frac{1}{2}{\left(x_{i}\right)}^{2}+\frac{1}{2}{\left(p_{i}\right)}^{2}-\gamma \right]V_{i}\left(R\right). \end{array}$$

Here, M is a diagonal mass matrix, and $V_{i}\left(R\right)$ is the potential energy of the electronic state *i*. Additionally, the kinematic momentum $P_{kin}$ is defined as

(6) $$\begin{array}{cc} P_{kin} & =P+\sum_{ij}^{F}\;x_{i}p_{j}d_{ij}\left(R\right) \end{array}$$

where $d_{ij}$ is the first-order non-adiabatic coupling vector.

The effective potential

(7) $$\begin{array}{cc} V_{\mathrm{eff}}\left(R\right) & =\sum_{i}^{F}\frac{1}{2}\left(x_{i}^{2}+p_{i}^{2}-2\gamma \right)V_{i}\left(R\right) \end{array}$$

is customarily rearranged into the symmetrized form

(8) $$\begin{array}{cc} V_{\mathrm{eff}}\left(R\right) & =\frac{1}{F}\sum_{i}^{F}V_{i}\left(R\right)+\frac{1}{F}\sum_{ij}^{F}\frac{1}{4}\left(x_{i}^{2}+p_{i}^{2}-x_{j}^{2}-p_{j}^{2}\right)\left(V_{i}\left(R\right)-V_{j}\left(R\right)\right), \end{array}$$

which enhances numerical stability in the trajectory propagation.

The classical equations of motion generated by the Hamiltonian of Equation (5) assume the form [113]

(9) $$\begin{array}{cc} {\dot{x}}_{i} & =p_{i}\frac{1}{F}\sum_{j}^{F}\left(V_{i}\left(R\right)-V_{j}\left(R\right)\right)+\sum_{j}^{F}x_{j}d_{ji}\left(R\right)\;\cdot \;\frac{P_{kin}}{M}\\ {\dot{p}}_{i} & =-x_{i}\frac{1}{F}\sum_{j}^{F}\left(V_{i}\left(R\right)-V_{j}\left(R\right)\right)+\sum_{j}^{F}p_{j}d_{ji}\left(R\right)\;\cdot \;\frac{P_{kin}}{M}\\ \dot{R} & =\frac{P_{kin}}{M}\\ {\dot{P}}_{kin} & =-\frac{\partial V_{\mathrm{eff}}\left(R\right)}{\partial R}+\frac{1}{2}\sum_{ij}^{F}\left(p_{i}p_{j}+x_{i}x_{j}\right)\left(V_{j}\left(R\right)-V_{i}\left(R\right)\right)d_{ji}\left(R\right), \end{array}$$

where ${\dot{x}}_{i}$, ${\dot{p}}_{i}$, $\dot{R}$, and ${\dot{P}}_{kin}$ are derivatives of $x_{i}$, $p_{i}$, R, and $P_{kin}$ with respect to t.

In the SQC/MMST simulations, the window function (which should not be confused with the window function of the DW representation) is employed for the initial sampling and final assignment of discrete electronic states [108,128]. The ZPE correction γ for the rectangular window function is applied in an ad hoc manner and is highly dependent on the specific system of interest [108,114,121]. In contrast, for the triangular window function, γ is determined based on geometric principles, exhibiting minimal dependence on the system. In practical applications, the triangular window function often outperforms the rectangular window function and is generally recommended for simulations [128]. Recent studies have rigorously derived triangular window functions for two-state systems [110,131], providing strong theoretical support for their adoption. The so-called triangle window function is defined as [129]

(10) $$\begin{array}{c} W_{i}\left(n=n_{1},\ldots n_{i},\ldots n_{f}\right)=w_{1}\left(n_{i}\right)\prod_{i\neq j}^{F}w_{0}\left(n_{i},n_{j}\right), \end{array}$$

where $n_{i}$ is the *i*th action variable

(11) $$w_{1}\left(n_{i}\right)=\{\begin{array}{cc} {\left(2-\gamma -n_{i}\right)}^{2-F} & \mathrm{for}\;1-\gamma <n_{i}<2-\gamma \\ 0 & \mathrm{otherwise} \end{array}$$

and

(12) $$w_{0}\left(n_{i},n_{j}\right)=\{\begin{array}{cc} 1 & \mathrm{for}\;n_{j}<2-2\gamma -n_{i}\\ 0 & \mathrm{otherwise} \end{array}$$

In other words, $W_{i}=1$ if the system is in the state *i*, and $W_{i}=0$ otherwise. Thus, the triangle window function recasts the action variables $n_{i}$ into the integers 0 or 1.

The action-angle sampling method produces initial electronic positions $x_{i}^{0}$ and momenta $p_{i}^{0}$, as follows:

(13) $$\begin{array}{c} x_{i}^{0}=\sqrt{2(n_{i}^{0}+\gamma )}\cos\theta ,\\ p_{i}^{0}=\sqrt{2(n_{i}^{0}+\gamma )}\sin\theta , \end{array}$$

where $n_{i}^{0}$ is an initial action variable sampled according to the triangle function, and θ∈[−π,π] is an angle variable used in the sampling. For the final assignment, the action variable $n_{i}$ can be obtained by

(14) $$\begin{array}{c} n_{i}=\frac{1}{2}{\left(x_{i}\right)}^{2}+\frac{1}{2}{\left(p_{i}\right)}^{2}-\gamma . \end{array}$$

Recently, Miller and Cotton proposed a trajectory-adjusted ZPE scheme, wherein each trajectory incorporates its own ZPE correction $\gamma_{i}$ [134]. Once the electronic coordinates $x_{i}^{0}$ and momenta $p_{i}^{0}$ are initially sampled, the trajectory-specific ZPE correction $\gamma_{i}$ is defined as

(15) $$\gamma_{i}=\frac{1}{2}{\left(x_{i}^{0}\right)}^{2}+\frac{1}{2}{\left(p_{i}^{0}\right)}^{2}-\delta_{ij},$$

where *j* is the adiabatic electronic state in the initial sampling. For two different points $\left(x_{1}^{0},p_{1}^{0}\right)$ and $\left(x_{2}^{0},p_{2}^{0}\right)$ within a triangular window, their ZPE corrections $\gamma_{1}$ and $\gamma_{2}$ are different according to Equation (15). Therefore, the value of $\gamma_{i}$ varies for each trajectory. Using this trajectory-specific $\gamma_{i}$, the action variable of the electronic DoF is defined as

(16) $$n_{i}=\frac{1}{2}{\left(x_{i}\right)}^{2}+\frac{1}{2}{\left(p_{i}\right)}^{2}-\gamma_{i}$$

during the trajectory propagation [134].

Meanwhile, the MMST Hamiltonian in the adiabatic representation is expressed in terms of the trajectory-adjusted $\gamma_{i}$ as follows:

(17) $$H=\frac{1}{2M}P_{kin}^{2}+\sum_{i}^{F}\left[\frac{1}{2}{\left(x_{i}\right)}^{2}+\frac{1}{2}{\left(p_{i}\right)}^{2}-\gamma_{i}\right]E_{i}\left(R\right).$$

More details on the trajectory-adjusted ZPE approach can be found in Ref. [134]. Recently, further interpretations and physical insights into the adjustable ZPE correction γ were presented [67,110,131,135,136].

### 2.2. DW Representation of TA PP Signals

The integral TA PP signal $I_{int}\left(\tau ,\omega_{pr}\right)$ can be obtained as a sum of non-rephasing (α=NR, $\xi_{\alpha }=1$) and rephasing (α=R, $\xi_{\alpha }=-1$) terms, each of which consists of the ground state bleach (GSB), stimulated emission (SE), and excited state absorption (ESA) contributions. $I_{int}\left(\tau ,\omega_{pr}\right)$ can be evaluated in terms of the third-order response functions $R_{\alpha k}\left(t_{3},t_{2},t_{1}\right)$ as follows [26,43,48,49]:

(18) Iint(τ,ωpr)∼Re∑α=R,NR∑k=0,I,II∫−∞∞dt∫0∞dt3∫0∞dt2∫0∞dt1×Epu(t+τ−t3−t2−t1)Epu(t+τ−t3−t2)Epr(t−t3)Epr(t)eiξαωput1eiωprt3Rαk(t3,t2,t1).

Here, k=0,I,II correspond to the GSB, SE, and ESA contributions; $E_{j}\left(t\right)$ and $\omega_{j}$ are the dimensionless envelopes of the pump (j=pu) and probe (j=pr) pulses; and τ is the time delay between them.

To evaluate the response functions $R_{\alpha k}\left(t_{3},t_{2},t_{1}\right)$, it is convenient to subdivide all molecular electronic states into three manifolds, 0, I, and II, which correspond to the ground, lower-lying, and upper-lying states. Then the molecular Hamiltonian of Equation (5) assumes the block-diagonal form,

(19) $$\begin{array}{cc} H= & \left(\begin{array}{ccc} H_{0} & H_{0I} & 0\\ H_{I0} & H_{I} & 0\\ 0 & 0 & H_{\mathrm{II}} \end{array}\right). \end{array}$$

Here, the Hamiltonians $H_{0}$, $H_{I}$, and $H_{\mathrm{II}}$—each of which can be represented as a sum of kinetic and potential parts—describe the (nonadiabatic) dynamic in the corresponding manifolds. $H_{0I}=H_{I0}^{†}$ are the inter-manifold coupling terms, which are relevant to the nonadiabatic couplings in the adiabatic representation. The rising and lowering TDM operators can also be recast in the block-diagonal form

(20) $$\begin{array}{cc} \mu^{↑}= & \left(\begin{array}{ccc} 0 & \mu_{0,I} & 0\\ 0 & 0 & \mu_{I,\mathrm{II}}\\ 0 & 0 & 0 \end{array}\right) \end{array},\;\;\;\begin{array}{cc} \mu^{↓}= & \left(\begin{array}{ccc} 0 & 0 & 0\\ \mu_{I,0} & 0 & 0\\ 0 & \mu_{\mathrm{II},I} & 0 \end{array}\right). \end{array}$$

As explained in Refs. [25,101], the TA PP signal of Equation (18) can be represented in the form suitable for on-the-fly simulations by performing a series of approximations. First, the quantum DW approximation is applied, according to which

(i) The pump and probe pulses are well temporally separated. Hence, the time delay τ between the pulses is (much) longer than the pump ($\tau_{pu}$) and probe ($\tau_{pr}$) pulse durations.

(ii) The pump and probe pulses are short on the nuclear dynamics timescale.

With these assumptions, the integral TA PP signal takes the DW form [25,101]

(21) $$\begin{array}{c} I_{int}\left(\tau ,\omega_{pr}\right)=\omega_{pr}\mathrm{Tr}\left[D\left(\omega_{pu}\right)\left(e^{iH_{0}\tau }W_{0}\left(\omega_{pr}\right)e^{-iH_{0}\tau }+e^{iH_{I}\tau }\left(W_{I}\left(\omega_{pr}\right)-W_{\mathrm{II}}\left(\omega_{pr}\right)\right)e^{-iH_{I}\tau }\right)\right], \end{array}$$

where

(22) $$\begin{array}{cc} D(\omega_{pu}) & =\int_{-\infty }^{\infty }dt_{2}^{\prime }\int_{0}^{\infty }dt_{1}E_{pu}\left(t_{2}^{\prime }\right)E_{pu}\left(t_{2}^{\prime }-t_{1}\right)e^{i\omega_{pu}t_{1}}e^{-iH_{I}t_{1}}\mu_{I,0}\rho_{0}e^{iH_{0}t_{1}}\mu_{0,I}+h.c. \end{array}$$

is the doorway operator; $\rho_{0}$ is the initial nuclear distribution in manifold 0, which is determined by either the thermal equilibrium or a particular vibrational level; and

(23) $$\begin{array}{cc} W_{0}(\omega_{pr})= & \int_{-\infty }^{\infty }dt\prime \int_{0}^{\infty }dt_{3}E_{pr}\left(t\prime \right)E_{pr}\left(t\prime +t_{3}\right)e^{i\omega_{pr}t_{3}}e^{iH_{0}t_{3}}\mu_{0,I}e^{-iH_{I}t_{3}}\mu_{I,0}+h.c.\\ W_{I}(\omega_{pr})= & \int_{-\infty }^{\infty }dt\prime \int_{0}^{\infty }dt_{3}E_{pr}\left(t\prime \right)E_{pr}\left(t\prime +t_{3}\right)e^{i\omega_{pr}t_{3}}\mu_{I,0}e^{iH_{0}t_{3}}\mu_{0,I}e^{-iH_{I}t_{3}}+h.c.\\ W_{\mathrm{II}}(\omega_{pr})= & \int_{-\infty }^{\infty }dt\prime \int_{0}^{\infty }dt_{3}E_{pr}\left(t\prime \right)E_{pr}\left(t\prime +t_{3}\right)e^{i\omega_{pr}t_{3}}\mu_{I,\mathrm{II}}e^{-iH_{\mathrm{II}}t_{3}}\mu_{\mathrm{II},I}e^{iH_{I}t_{3}}+h.c. \end{array}$$

are the window operators.

Then, a series of classical DW approximations is applied.

(iii) The DW operators $D(\omega_{pu})$ and $W_{k}\left(\omega_{pr}\right)$ become functions of the nuclear coordinates R and momenta P, $D(\omega_{pu},R,P)$, and $W_{k}\left(\omega_{pr},R,P\right)$.

(iv) Radiative transitions are treated within the classical Condon approximation [75,137].

(v) The initial vibrational distribution $\rho_{0}$ in the doorway function (22) is replaced by the Wigner distribution $\rho_{g}^{Wig}\left(R,P\right)$ [138].

(vi) The trace Tr[…] in Equation (21) is evaluated by Monte Carlo averaging $\langle \ldots \rangle$ over classical initial conditions.

(vii) The Heisenberg propagators in Equation (21) are replaced by the evolution along (quasi) classical trajectories R(T),P(T).

For converting these approximations into operational expressions and simulation protocols, we denote electronic states in manifolds 0, I, and II as |g〉, |e〉, and |f〉; potential energy functions in these states as $V_{g}\left(R\right)$, $V_{e}\left(R\right)$, and $V_{f}\left(R\right)$; transition frequencies as $U_{eg}\left(R\right)=V_{e}\left(R\right)-V_{g}\left(R\right)$ and $U_{fe}\left(R\right)=V_{f}\left(R\right)-V_{e}\left(R\right)$; and matrix elements of TDMs as $\mu_{ge}\left(R\right)=\langle g|\mu_{0,I}|e\rangle$ and $\mu_{fe}\left(R\right)=\langle f|\mu_{\mathrm{II},I}|e\rangle$. Then, approximations (iii) and (iv) yield [75,137]

(24) $$\langle g|e^{iH_{0}t_{3}}\mu_{0,I}e^{-iH_{I}t_{3}}|e\rangle \approx e^{iU_{ge}\left(R\right)t_{3}}\mu_{ge}\left(R\right),$$

(25) $$\langle f|e^{iH_{\mathrm{II}}t_{3}}\mu_{\mathrm{II},I}e^{-iH_{I}t_{3}}|e\rangle \approx e^{iU_{fe}\left(R\right)t_{3}}\mu_{fe}\left(R\right)$$

and we arrive at the final quasiclassical DW expression for the integral TA PP signal, as follows:

(26) $$\begin{array}{cc} I_{int}(\tau ,\omega_{pr})= & I_{int}^{GSB}\left(\tau ,\omega_{pr}\right)+I_{int}^{SE}\left(\tau ,\omega_{pr}\right)+I_{int}^{ESA}\left(\tau ,\omega_{pr}\right),\\ I_{int}^{GSB}(\tau ,\omega_{pr})= & \omega_{pr}\sum_{e}\int dR_{g}dP_{g}D\left(\omega_{pu},R_{g},P_{g}\right)W_{0}\left(\omega_{pu},R_{g}\left(\tau \right),P_{g}\left(\tau \right)\right),\\ I_{int}^{SE}(\tau ,\omega_{pr})= & \omega_{pr}\sum_{e}\int dR_{g}dP_{g}D\left(\omega_{pu},R_{g},P_{g}\right)W_{I}\left(\omega_{pu},R_{e}\left(\tau \right),P_{e}\left(\tau \right)\right),\\ I_{int}^{ESA}(\tau ,\omega_{pr})= & -\omega_{pr}\sum_{e}\int dR_{g}dP_{g}D\left(\omega_{pu},R_{g},P_{g}\right)W_{\mathrm{II}}\left(\omega_{pu},R_{e}\left(\tau \right),P_{e}\left(\tau \right)\right). \end{array}$$

Here, $R_{g}$ and $P_{g}$ represent the initial nuclear coordinates and momenta in the electronic ground state sampled according to the Wigner distribution $\rho_{g}^{Wig}\left(R_{g},P_{g}\right)$, $R_{g}\left(\tau \right)$ and $P_{g}\left(\tau \right)$ denote the nuclear coordinates and momenta propagated in the electronic ground state up to t=τ, and $R_{e}\left(\tau \right)$ and $P_{e}\left(\tau \right)$ denote the nuclear coordinates and momenta propagated up to t=τ in manifold I of the lower-lying excited electronic states. The quasiclassical doorway function reads,

(27) $$\begin{array}{cc} D(\omega_{pu},R_{g},P_{g})= & |\mu_{ge}\left(R_{g}\right){|}^{2}E_{pu}^{2}\left(\omega_{pu}-U_{eg}\left(R_{g}\right)\right)\rho_{g}^{Wig}\left(R_{g},P_{g}\right), \end{array}$$

and the quasiclassical window functions are defined as

(28) $$\begin{array}{cc} W_{0}(\omega_{pu},R_{g}\left(\tau \right),P_{g}\left(\tau \right))= & \sum_{e}{\left|\mu_{ge}\left(R_{g}\left(\tau \right)\right)\right|}^{2}E_{pr}^{2}\left(\omega_{pr}-U_{eg}\left(R_{g}\left(\tau \right)\right)\right),\\ W_{I}(\omega_{pu},R_{e}\left(\tau \right),P_{e}\left(\tau \right))= & |\mu_{ge(\tau )}\left(R_{e}\left(\tau \right)\right){|}^{2}E_{pr}^{2}\left(\omega_{pr}-U_{e(\tau )g}\left(R_{e}\left(\tau \right)\right)\right),\\ W_{\mathrm{II}}(\omega_{pu},R_{e}\left(\tau \right),P_{e}\left(\tau \right))= & \sum_{f}{\left|\mu_{e(\tau )f}\left(R_{e}\left(\tau \right)\right)\right|}^{2}E_{pr}^{2}\left(\omega_{pr}-U_{fe(\tau )}\left(R_{e}\left(\tau \right)\right)\right). \end{array}$$

Here, $E_{pu}\left(\omega \right)$ and $E_{pr}\left(\omega \right)$ are the Fourier transforms of $E_{pu}\left(t\right)$ and $E_{pr}\left(t\right)$, and the notion e(τ) means that a trajectory initiated in an excited state *e* may jump into another electronic state.

The above derivations have been made under the assumption that nonadiabatic coupling operators $H_{0}I=H_{I}^{†}0$ can be neglected on the timescale of interest. Once the internal conversion e(τ)→g is allowed, Equation (21) remains valid, but the window functions $W_{k}$ defined per Equation (28) have to be replaced by the new window functions ${\bar{W}}_{k}$, which are defined in terms of the original window functions as follows [101]:

(29) $${\bar{W}}_{0}=W_{0},$$

(30) $${\bar{W}}_{I}=\left[\begin{array}{c} W_{I},\;\;\;\;\;\;\;\;\;\;\;\;\mathrm{if}\;\mathrm{trajectory}\;\mathrm{stays}\;\mathrm{within}\;I\\ -W_{0},\;\;\;\mathrm{if}\;\mathrm{trajectory}\;\mathrm{jumps}\;\mathrm{from}\;I\;\mathrm{to}\;0, \end{array}\right.$$

(31) $${\bar{W}}_{\mathrm{II}}=\left[\begin{array}{c} W_{\mathrm{II}},\;\;\;\;\;\;\;\;\;\mathrm{if}\;\mathrm{trajectory}\;\mathrm{stays}\;\mathrm{within}\;I\\ 0,\;\;\;\mathrm{if}\;\mathrm{trajectory}\;\mathrm{jumps}\;\mathrm{from}\;I\;\mathrm{to}\;0. \end{array}\right.$$

If a trajectory jumps from manifold I back to the ground state, then e(τ) changes to *g* in Equation (30). Owing to the e(τ)→g internal conversion, two GSB contributions arise: the cold and the hot. The cold contribution is the conventional GSB signal, which reveals the nuclear wavepacket on the electronic ground state. It is described by the window function $W_{0}$ of Equation (29). The hot contribution is the internal-conversion-induced GSB signal that reveals the manifold I trajectory, which jumps to the electronic ground state after the e(τ)→g internal conversion. This trajectory contributes to the GSB signal with a minus sign, $-W_{0}$ in Equation (30) [101]. In the SQC/MMST simulations, the DW functions are straightforwardly calculated by “binning” trajectories to the windows corresponding to the currently occupied electronic states [129].

### 2.3. Computational Details

**Figure 1:**
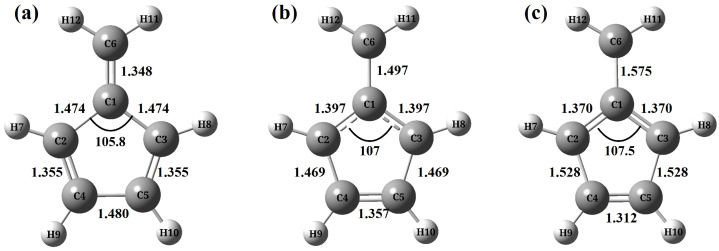
Optimized equilibrium structures of fulvene in (**a**) $S_{0}$, (**b**) $S_{1}$, and (**c**) CI.

The optimized structures of fulvene are shown in Figure 1. The $S_{0}$ minimum was optimized at the B3LYP/6-31G(d) level by using the Gaussian 16 package [139]. In the SQC/MMST simulations, two state-averaged CASSCF(6,6) method with the basis set 6-31G(d) was employed in the MOLPRO2022 package [140]. Initial nuclear coordinates and momenta were sampled according to the Wigner distribution of the lowest vibrational level on the ground electronic state [141]. Initial sampling and final assignments of electronic states were performed by using the symmetrical triangle window function of Equation (10), while coordinates and momenta of mapping electronic degrees of freedom were generated according to the action-angle distribution of Equation (13). The time steps were fixed at 0.2 fs and 0.002 fs for nuclear and mapping electronic motions, respectively. All dynamics calculations were carried out with the JADE package [115,141].

For fulvene, manifold I contains a single state $S_{1}$, while manifold II consists of four states, $S_{2}$–$S_{5}$. A total of 100 trajectories were initiated on the $S_{1}$ state and propagated for 600 fs in manifold I to simulate the hot GSB, SE, and ESA signals. Another 100 trajectories were initiated on the $S_{0}$ state and propagated for 600 fs to simulate the cold GSB signal. To evaluate ESA signals, snapshots from every trajectory were taken to obtain vertical excitation energies and TDMs for the states of manifold II.

Gaussian pump and probe pulses were chosen, $E_{a}\left(t\right)=\exp\left\{-{\left(t/\tau_{a}\right)}^{2}\right\}$ and $E_{a}\left(\omega \right)=\exp\left\{-{\left(\omega \tau_{a}\right)}^{2}/4\right\}$, with $\tau_{a}=2$ fs. This gives a pulse bandwidth of 1.04 eV (full width at half maximum).

## 3. Results and Discussion

**Figure 2:**
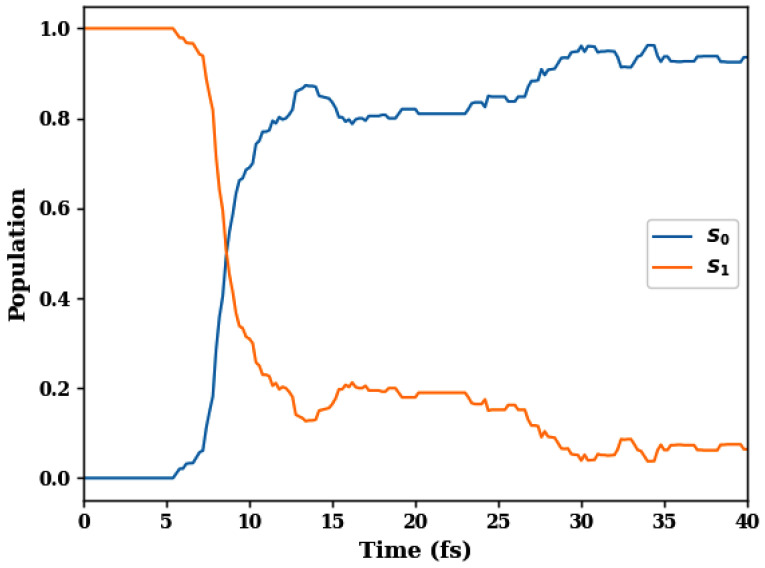
Time-dependent populations of the $S_{0}$ and $S_{1}$ states of fulvene.

To set up the stage, we begin with the populations of the $S_{0}$ and $S_{1}$ states of fulvene, which are shown in Figure 2. These populations correlate with those obtained by the numerically accurate AIMS method [118,119,120,121] as well as with those computed by the SQC/MMST method [121]. No decay of the $S_{1}$ population occurs within the first 5 fs, during which the wavepacket travels from the Franck–Condon to the CI region. Then, the $S_{1}$ population decreases rapidly, reaching half of its initial value at 9 fs and less than 20% within 10–15 fs. After that, a weak-amplitude population recurrence occurs within 15–25 fs. This flat recurrence is caused by the torsional motions, which bring the wavepacket back to the PES crossing each 20–25 fs. After the recurrence, the $S_{1}$ population remains small, but its quenching slows down considerably.

**Figure 3:**
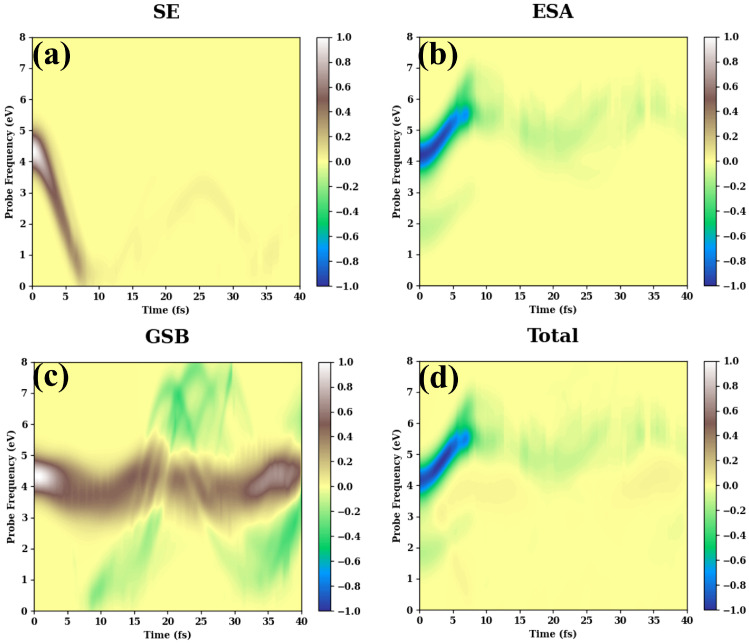
Normalized SE (**a**), ESA (**b**), GSB (**c**), and total (**d**) integral TA PP spectra of fulvene vs. pump-probe delay τ and the probe carrier frequency $\omega_{pr}$. The carrier frequency of the pump pulse, $\omega_{pu}=4.39$ eV, is in resonance with the $S_{1}$ state.

Figure 3a–c show the SE, ESA, GSB contributions to the integral TA PP spectrum, while panel (d) displays the total TA PP spectrum. The spectra are initiated with the pump pulse with $\omega_{pu}=4.39$ eV, which is in resonance with the $S_{0}-S_{1}$ transition (see Table A1). Note that all observables of the present section are evaluated at the SA6-CASSSCF level. The SE signals obtained at the SA2-CASSCF level are given in Appendix A. These two levels of the electronic structure theory give very similar results. The long-time TA PP spectra are also shown in Appendix A.

At τ=0, the SE spectrum is concentrated around $\omega_{pu}$, as expected. Within the first 10 fs, it rapidly moves to the red. This redshift is accompanied by the significant loss of intensity. Then $I_{int}^{SE}\left(\tau ,\omega_{pr}\right)$ exhibits a pronounced recurrence around τ≈25 fs, but the overall intensity of the spectrum after 10 fs is roughly an order of magnitude lower than the initial intensity. This behavior correlates with the $S_{1}$ population evolution in Figure 2. However, the $S_{1}$ population recurrence tells us nothing about the details of the wavepacket motion on $S_{1}$. The SE signal, on the contrary, demonstrates that a fraction of the wavepacket returns almost to the initial position in the Franck–Condon region. This indicates that the SE signal can be used as an informative observable containing more information than the traditional excited-state population. Note that the low-intensity high-amplitude oscillations in $I_{int}^{SE}\left(\tau ,\omega_{pr}\right)$ last for several hundreds of femtoseconds (see Appendix A), indicating a partially reversible wavepacket motion at the CI. This kind of behavior is highly unusual for CI systems, which normally exhibit irreversible ultrafast population transfer [1,2].

The ESA contribution is shown in Figure 3b. At short times, $I_{int}^{ESA}\left(\tau ,\omega_{pr}\right)$ has two components: the upper high-intensity component starting at $\omega_{pr}\approx 4.2$ eV and the lower low-intensity component starting at $\omega_{pr}\approx 2$ eV. These two branches of the ESA spectrum reveal the $S_{1}-S_{4}/S_{5}$ and the $S_{1}-S_{2}$ transitions, respectively (see Appendix A). Initially, both ESA components move to the higher energy domain and reach their maxima around 10 fs. Then, the intensity of the upper component decreases rapidly, exhibits a minimum around 20 fs, and continues oscillatory evolution at longer times. The lower component vanishes after 10 fs on the intensity scale of the figure. Both SE and ESA signals reflect the same wavepacket motion on the $S_{1}$ state. They differ in the “spectator states” (manifold 0 for SE and manifold II for ESA), which are used for the detection of the signals. It is not surprising, therefore, that the SE and ESA signals behave similarly. More precisely, the spectra in panels (a) and (b) look like mirror images of each other: while the SE spectrum moves to the red, reaches a minimum, exhibits a maximum, and keeps oscillating at much lower intensity, the ESA spectrum moves to the blue, reaches a maximum, exhibits a minimum, and keeps oscillating at a much lower intensity. This mirror behavior indicates that the energy gap between the states contributing to the SE (ESA) spectrum initially decreases (increases) with τ.

Figure 3c shows the GSB signal, which is composed of the cold (positive) and hot (negative) components. The cold spectrum exhibits undamped oscillations with a ∼2 eV amplitude, ∼20 fs period, and ∼4 eV baseline. The spectrum is dominated by the $S_{0}-S_{1}$ transitions in the Franck–Condon region (see Appendix A). The hot spectrum emerges at ∼10 fs (which correlates with the initial decay of the $S_{1}$ population in Figure 2) and shows high-amplitude (∼7 eV) oscillations with a period of ∼20 fs around the baseline of ∼4 eV. Such a large oscillation amplitude of the hot spectrum is caused by the excess kinetic energy acquired by the wavepacket in the course of its CI-driven internal conversion. The same baseline of 4 eV is a signature of the dominant role of the $S_{0}-S_{1}$ transitions in the formation of both cold and hot signals. Similar oscillation periods of the cold and hot components seem to be a remarkable feature of fulvene, caused by the fact that various nuclear motions in this molecule have similar periods around 20 fs.

The total TA PP signal is shown in Figure 3d. It is dominated by the ESA contribution. This is a consequence of the large values of TDMs to higher-lying excited states of manifold II.

The above discussions clearly show the close correspondence between the TA PP signals in Figure 3 and the population dynamics in Figure 2. However, it is even more important to ask whether it is possible to establish correlation between the spectral patterns in the TA PP signals and the key molecular motions in the nonadiabatic dynamics. For this purpose, several critical structures in the nonadiabatic dynamics were optimized at the SA2-CASSCF(6,6)/6-31G(d) level, as shown in Figure 1.

The $S_{0}$ equilibrium in Figure 1a shows an exocyclic bond with a single-bond–double-bond alternation. For the $S_{1}$ equilibrium (b), however, the alternation is reversed and reveals a biradical character [126]. As shown in Figure 1c, the $S_{0}S_{1}$ conical intersection (CI) geometry closely resembles that of the $S_{1}$ minimum. However, a detailed examination of their internal coordinates reveals that the $S_{1}$ minimum lies between the $S_{0}$ minimum and the $S_{0}S_{1}$ CI. This suggests that rapid nonadiabatic decay is likely to occur following the initial vibrational relaxation to the $S_{1}$ minimum. This conclusion is supported by the fact that all bond distances at the S1 minimum fall between their corresponding values at the $S_{0}$ minimum and the $S_{0}S_{1}$ CI. Specifically, from the $S_{0}$ minimum to the $S_{1}$ minimum and further to the $S_{0}$$S_{1}$ CI, the C1–C6, C2–C4, and C3–C5 bond distances increase monotonically, while the C1–C2 and C1–C3 bond distances decrease monotonically. Additionally, the C2–C1–C3 angle is smaller at the $S_{0}$ equilibrium geometry but larger near the $S_{0}S_{1}$ CI. Therefore, the wavepacket motion from the Franck–Condon (FC) region to the $S_{1}$ minimum and further toward the CI region involves the extension of the C2–C4 and C3–C5 bonds, the contraction of the C1–C2 and C1–C3 bonds, and a decrease in the C2–C1–C3 angle (see Figure 1). These findings are consistent with previous studies [126].

**Figure 4:**
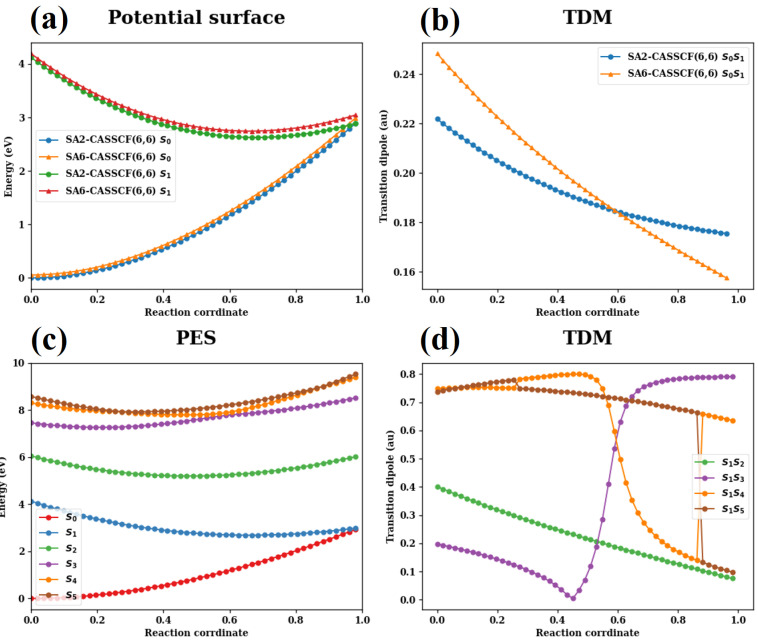
(**a**) Linear interpolated PESs of $S_{0}$ and $S_{1}$ between $S_{0}$ minimum and $S_{0}/S_{1}$ CI obtained at SA2-CASSCF(6,6)/6-31G(d) and SA6-CASSCF(6,6)/6-31G(d) levels. (**b**) Linear interpolated TDMs from $S_{0}$ to $S_{1}$ between $S_{0}$ minimum and $S_{0}/S_{1}$ CI obtained at SA2-CASSCF(6,6)/6-31G(d) and SA6-CASSCF(6,6)/6-31G(d) levels. (**c**) Linear interpolated PESs from $S_{0}$ to $S_{5}$ between $S_{0}$ minimum and $S_{0}/S_{1}$ CI. (**d**) Linear interpolated TDMs from $S_{1}$ to $S_{2}-S_{5}$ between $S_{0}$ minimum and $S_{0}/S_{1}$ CI.

The linear-interpolated reaction pathways between the optimized $S_{0}$ minimum and $S_{0}/S_{1}$ CI and the relevant TDMs obtained by the single point calculations at both SA2-CASSCF(6,6)/6-31G(d) and SA6-CASSCF(6,6)/6-31G(d) levels are presented in Figure 4. Both levels of electronic structure theory give very similar results for potential energy profiles and TDMs. It is thus safe to use any of them for the simulation of spectroscopic signals. This is corroborated by the comparison of Figure 3 and its counterpart in Appendix A.

Along the interpolated pathways that are driven by the above reactive coordinates and shown in Figure 4a,c, no barrier exists between the Franck–Condon region and the CI seam. In the Franck–Condon region, a large gradient is observed. Clearly, there is a $S_{1}$ minimum in the pathway toward the CI, and this minimum lies lower than the CI. As a result, the so-called sloped CI is created [126]. Based on the PES profiles, the system is expected to transition from the FC region to the S1 minimum and then proceed to the CI region. This observation is consistent with the geometric characteristics of these critical geometries.

As illustrated in Figure 4b, TDM between the $S_{0}$ and $S_{1}$ states decreases along the reaction pathway. Figure 4d reveals electronic transitions from the $S_{1}$ state to higher-lying states ($S_{2}$, $S_{3}$, $S_{4}$, and $S_{5}$). Notably, the $S_{1}-S_{4}$ and $S_{1}-S_{5}$ TDMs are larger than the $S_{0}-S_{1}$ TDM in the Franck–Condon region, which explains why the ESA signals are stronger than the SE signals. Interestingly, several abrupt changes in TDM are observed along the reaction pathways, attributed to the existence of PES crossings. When two states cross, the sudden switch between their adiabatic electronic wavefunctions leads to abrupt changes in physical observables, such as TDMs. In this context, such sudden TDM changes serve as indicators of conical intersections. More specifically, in the current system, the rise in the $S_{1}-S_{3}$ TDM and the drop in the $S_{1}-S_{4}$ TDM are associated with the $S_{3}/S_{4}$ surface crossing. Additionally, we observed two geometries where the TDM values between the $S_{1}-S_{4}$ and $S_{1}-S_{5}$ transitions exchange, indicating the presence of two $S_{4}/S_{5}$ PES crossing points.

**Figure 5:**
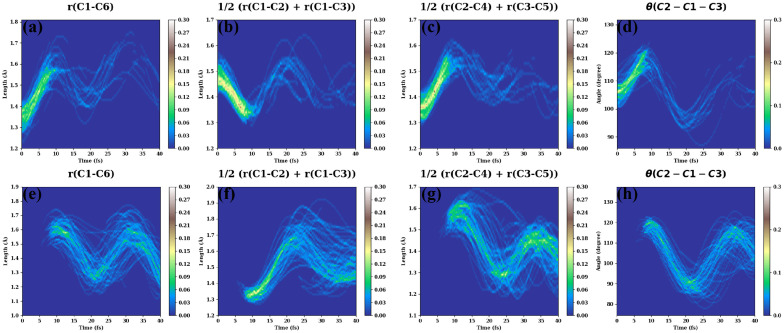
Normalized time-dependent distributions of C1–C6 bond lengths (**a**,**e**), C1–C2 and C1–C3 symmetric stretches (**b**,**f**), C2–C4 and C3–C5 symmetric stretches (**c**,**g**), and C2–C1–C3 bendings (**d**,**h**). The upper panels are evaluated for the first excited state $S_{1}$, while the lower panels are evaluated for $S_{0}$.

Ab initio simulations permit one to explore how the time evolution of TA PP signals is correlated with the dynamics of specific molecular bond lengths and angles. The results are presented in Figure 5. Figure 5a shows how the C1–C6 bond distance evolves in time on the $S_{1}$ (a) and $S_{0}$ (e) states. In the first 10 fs, elongation of the C1–C6 bond is observed. When the C1–C6 distance reaches 1.5–1.7 Å, some trajectories jump back to the ground state, as is clear from Figure 5a,e. The C1–C6 bond displays pronounced oscillations on the $S_{1}$ state. Starting from 7–10 fs, a large-amplitude stretching motion of the C1–C6 bond emerges also on the ground state. Evolutions of the bond distances in Figure 5b,c,f,g on the $S_{1}$ state also exhibit qualitatively similar behaviors. Figure 5d,h display evolutions of the bond angle C2–C1–C3 on the excited and ground electronic states, respectively. In Figure 5d, the C2–C1–C3 angle increases during the first 10 fs. When the angle reaches 110°–120°, only a few trajectories remain on $S_{1}$. Afterward, the remaining trajectories produce high-amplitude oscillations of the C2–C1–C3 angle. There are no trajectories on the $S_{0}$ state before 10 fs (Figure 5h). At ≈10 fs, abundant ground-state trajectories emerge for the C2–C1–C3 angles around 110°–120°. At longer times, a large amplitude of the C2–C1–C3 bending was observed. Note that the evolution of the C2–C1–C3 angle (Figure 5d,h) is synchronous with the C1–C6 stretching motion (Figure 5a,e). Such unusually fast bending motion can be explained by the fact that the C1–C6 stretching motion and the C2–C1–C3 bending motion are strongly coupled with each other since both involve the C1 atom. In contrast, other motions (e.g., the C4–C5 stretching, see Appendix A) are only weakly involved in the dynamic propagation, as they either show small oscillation amplitudes or different oscillation periods.

Interestingly, the nuclear motions on $S_{0}$ are more erratic than those on $S_{1}$. This is manifested in a larger spread of trajectories in the lower panels in comparison with that in the upper panels. The reason is that internal conversion produces large-amplitude wavepackets, which experience vibrational energy redistribution induced by the mode–mode couplings.

By the combination of the above discussions, the relationship between the population dynamics, the key molecular motions, and the TA PP signals is well established. As the carrier frequency of the pump pulse is resonant with the $S_{0}-S_{1}$ transition, the nonadiabatic dynamics of fulvene start from the first excited state. In the Franck–Condon region, the large gradient of the $S_{1}$ state drives ultrafast nuclear motions, leading to pronounced stretching (C1–C6, C1–C2/C1–C3, C2–C4/C3–C5) and bending (C2–C1–C3) motions. Following these motions, the system first arrives in the $S_{1}$ minimum and then moves close to the CI on the excited state. This motion also leads to the TDM decrease (Figure 4). As a consequence, the SE signal moves to the low-frequency domain quickly, which is consistent with the time scale of the aforementioned nuclear motions along the internal coordinates. In addition, all trajectories follow similar propagation patterns, and no dispersion is observed. Thus, the SE signal does not show bifurcation in the early stage of the dynamics. Similar patterns were observed in the ESA signal. The only difference is that the ESA signal moves to the higher-energy domain because energy gaps between the $S_{1}$ state and the higher-lying excited electronic states responsible for the relevant transitions increase with time.

When the CI is reached, many trajectories jump back to the ground state, resulting in significant population transfer. Hence, both the SE and ESA signals suddenly become weaker at ∼7–10 fs, which directly reflects the ultrafast nonadiabatic transitions. After that, a few trajectories stay in the excited state for a rather long time. As the $S_{1}$ minimum is lower than the CI and the shape of the CI is sloped, these trajectories oscillate near the $S_{1}$ minimum. Due to the lack of dissipation, these oscillations endure for quite a long time and induce the oscillatory patterns in the weak SE and ESA signals (see Appendix A). Whenever the trajectories access the CI, there is a minor fluctuation of the electronic population due to the nonadiabatic transition, which is consistent with the population dynamics in Figure 2. As some trajectories jump back to the ground state, they create the GSB hot signal. Here, the molecular vibrations are highly excited by the excessive energy emerged after internal conversion. This leads to diversity in the trajectory propagation patterns and results in quite erratic hot GSB signals. Due to the small system size and lack of dissipation, these signals show large-amplitude oscillations, and their periods are consistent with the vibrational periods of the few key coordinates, such as the C1–C6 stretching motion. At the same time, the mode–mode coupling is also responsible for the spreading of trajectories, producing a broad hot GSB signal. Overall, the TA PP signals provide clear fingerprints, which allow us to monitor the population decay and identify the key tuning modes in the nonadiabatic dynamics.

In addition, evolutions of the asymmetric stretching modes are given in Appendix A. The modes exhibit oscillations with a ∼20 fs period and different initial phases. As a result, these modes produce characteristic pulsating wavepackets, which are symmetric with respect to positive and negative elongations. There are several demonstrations on how to extract frequencies of the coupling modes from the femtosecond spectra [142,143,144]. In the present case of fulvene, however, both coupling and tuning modes exhibit oscillations with a period of ∼20 fs. Hence, we cannot unambiguously obtain information on the coupling modes from the TA PP spectra.

**Figure 6:**
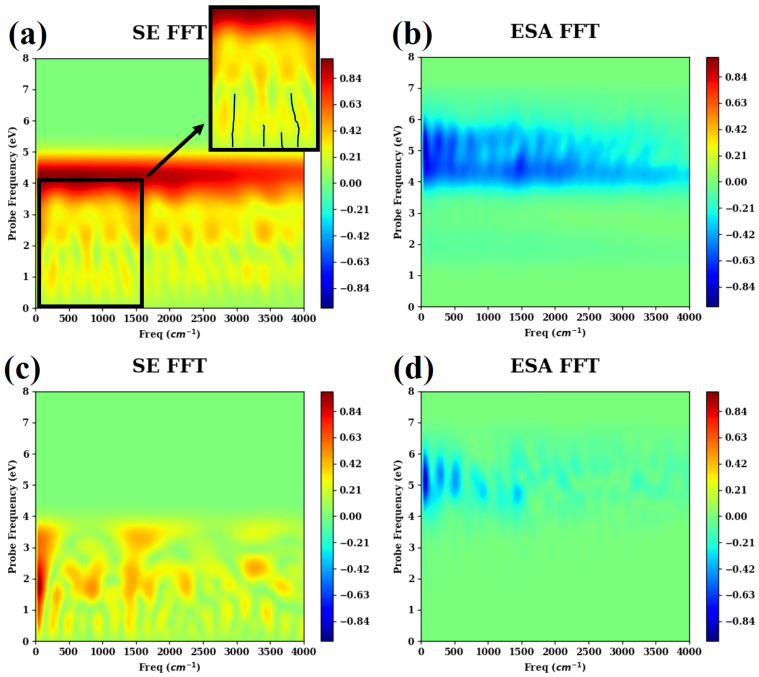
Fourier transforms of the normalized TA PP spectra of fulvene with respect to the population time τ: $I_{int}^{SE}\left(\Omega ,\omega_{pr}\right)$ (**a**,**c**) and $I_{int}^{ESA}\left(\Omega ,\omega_{pr}\right)$ (**b**,**d**). The upper spectra are computed by integrating from 0 to 600 fs, while the lower spectra are evaluated by integrating from 10 to 600 fs. In addition, the tiny black curves in the inset of (**a**) show the splitting of the board peaks.

Unfortunately, experimental femtosecond TA PP signals of fulvene are not available. However, the TA PP spectra can be used as important theoretical observables containing useful information on the CI-driven wavepacket dynamics. This is further illustrated by Figure 6, which shows Fourier transforms of the SE (a,c) and ESA (b,d) spectra with respect to the population time τ,

(32) $$I_{int}^{k}\left(\Omega ,\omega_{pr}\right)=\int d\tau e^{i\Omega \tau }I_{int}^{k}\left(\tau ,\omega_{pr}\right)$$

(k=SE,ESA). These analyses are inspired by the successful use of beating map for disentangling electronic 2D spectra [145,146]. Let us consider the SE spectrum $I_{int}^{SE}\left(\Omega ,\omega_{pr}\right)$ (a) first. Its value around $\omega_{pr}∼4$ eV gives a broad featureless spectrum along Ω, which is a mere indication of the short lifetime of the system in $S_{1}$. If we move to $\omega_{pr}\;∼$2.5 eV, we see a series of relatively broad peaks that are dominated by the high-intensity peak around Ω≈ 1500–1700 cm^−1^, which yields a 20 fs period, which dominates all temporal evolutions of the spectra. The peak widths indicate the significance of anharmonic effects produced by nonadiabatic couplings. If we inspect the spectrum around $\omega_{pr}∼2.5$ eV, we observe splitting of the majority of the broad peaks into pairs of narrower subpeaks. This is a clear signature of the mode–mode coupling. The ESA spectrum $I_{int}^{ESA}\left(\Omega ,\omega_{pr}\right)$ (b) also reveals multiple broad peaks dominated by the one at Ω≈ 1500–1700 cm^−1^. The peaks do not exhibit the doublet splitting, which is characteristic of the SE peaks, owing to the larger number of transitions contributing to the ESA spectra. The exclusion of the first 10 fs from the Fourier transform (panels c, d) removes the most intense part of the TA PP, and the corresponding $I_{int}^{SE}\left(\Omega ,\omega_{pr}\right)$ and $I_{int}^{ESA}\left(\Omega ,\omega_{pr}\right)$ exhibit much simpler peak structures featuring a well-separated Ω≈ 1500–1700 cm^−1^ peak.

The totally symmetric modes/motions are very important, as they are the so-called “tuning” modes/coordinates in the nonadiabatic dynamics. Both the SE and ESA signals contain information on these tuning modes, because the two signals project the same excited-state wavepacket motion to the electronic ground state (SE) and higher-lying excited states (ESA). Here, we try to discuss the correspondence between the spectra and molecular motions in detail, although such analyses are highly qualitative.

All optically active motions such as C1–C6, C1–C2/C1–C3, and C2–C4/C3–C5 stretching motions, display the oscillation periods around 20 fs. The SE and ESA peaks around 1500–1700 cm^−1^ should reflect these motions. However, due to the low-frequency resolution, it is not easy to distinguish these motions with similar periods in Figure 6. The evolution features show that the C2–C1–C3 bending motion contains the fast oscillation components in this time domain, which should also contribute to the SE and ESA peaks around 1500–1700 cm^−1^. In addition, the slow component of the C2–C1–C3 bending motion with the oscillation period at 48 fs should be relevant to the SE and ESA peak at 700 cm^−1^ (see Appendix A). The slow components of several stretching motions (see Appendix A) also belong to a similar frequency domain. This indicates the existence of strong mode–mode couplings. There are also low-frequency peaks (100–300 cm^−1^) that cannot be assigned to specific totally symmetric modes due to the mismatch of the frequencies. They reveal pronounced anharmonicities and mode–mode couplings in fulvene. As a consequence, the low-frequency modes should be excited, resulting in the corresponding oscillations. At the same time, the Fourier transformation of the stretching motions also contains such low-frequency components (see Appendix A). These may be relevant to the low-frequency parts in the spectral analyses. We also point that the Fourier transformation is not very trustable for low-frequency modes. For example, 100 cm^−1^ corresponds to a period of 330 fs, which gives less than two periods on the employed 600 fs simulation timescale.

We wish to emphasize that the current analyses based on the Fourier transformation only give qualitative understanding because the time duration of the current nonadiabatic dynamics results in a rather low resolution in the frequency domain. In addition, it is not fully suitable to rely on the normal mode picture to analyze the molecular motion in the current nonadiabatic dynamics because of excessive energies, high anharmonicities, strong mode–mode couplings, and highly vibrational excitations. Therefore, the vibrational motions of internal coordinates are employed in the current qualitative analyses, which all show the fast and slow components simultaneously.

## 4. Conclusions

By combining the SQC/MMST dynamics and the DW representation, we build an efficient protocol for the on-the-fly simulation of femtosecond TA PP signals. The protocol can readily be extended to electronic 2D and other third-order spectroscopic signals.

In the present work, the protocol has been employed to simulate integral TA PP spectrum of fulvene. On the one hand, the SE and ESA contributions to the TA PP spectrum reveal the characteristic times of the CI-driven $S_{1}-S_{2}$ internal conversion, which can be extracted from the $S_{1}$ population dynamics. On the other hand, analyses of the GSB-cold, GSB-hot, SE, and ESA components of the TA PP spectrum give richer and much more comprehensive information on the nuclear motions contributing to the nonadiabatic dynamics. This indicates that ultrafast spectroscopic signals can also be considered as information-rich purely theoretical observables, which are complementary to the standard set of dynamic observables that includes evolutions of populations, bond lengths, and bond angles. Taken together, the observables contain information on the peculiarities of the wavepacket motion on the ground and lower-lying excited electronic states, mode-coupling effects, and the leading active modes responsible for the CI-driven $S_{1}-S_{0}$ internal conversion in fulvene. For instance, oscillations of the TA PP signals in the time domain can be attributed to the symmetric stretching and bending modes on the $S_{1}$ and $S_{0}$ states, and amplitudes of these oscillations provide information on the relative shifts of the corresponding PESs.

Fulvene is a molecular representative of Tully’s third model [117]. Recently, Worth and coworkers compared electronic populations of three Tully’s molecules predicted by the fewest switches surface hopping method, variational multi-configuration Gaussian (vMCG) method, and (numerically accurate) multi-configuration time-dependent Hartree (MCTDH) method [147]. All calculations employed the linear vibronic coupling (LVC) Hamiltonians developed for the three molecules. As was demonstrated in our work, electronic populations are just the most conventional observables, while much deeper and stringent tests would require a comparison of nonlinear spectroscopic signals. Obviously, the construction of LVC Hamiltonians for higher-lying excited states is a difficult and not fully microscopically justified task, but the available LVC Hamiltonians can be used for the evaluation and comparison of SE and GSB contributions to all third-order spectroscopic signals. The next step may involve the testing of on-the-fly and LVC-based signals evaluated by different quantum and quasiclassical methods. Our work can be regarded as a first step in this direction, and the DW methodology can become an efficient tool for the evaluation of all spectroscopic signals.

## Figures and Tables

**Figure A1:**
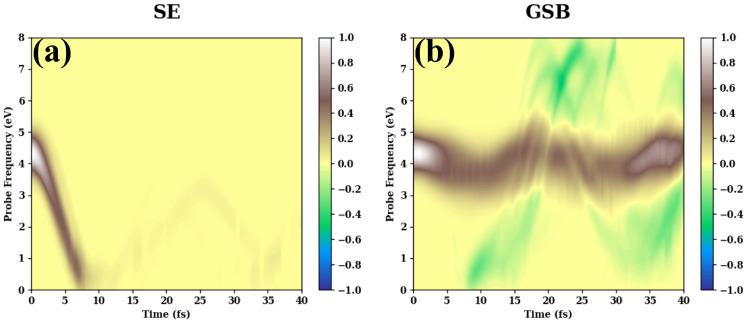
Normalized (**a**) SE signal and (**b**) GSB signal of fulvene versus the delay time τ and the probe signal $\omega_{pr}$ obtained at the SA2-CASSCF(6,6) level of electronic structure theory. The pump-pulse frequency $\omega_{pu}=4.39$ eV is resonant with the $S_{1}$ state.

**Figure A2:**
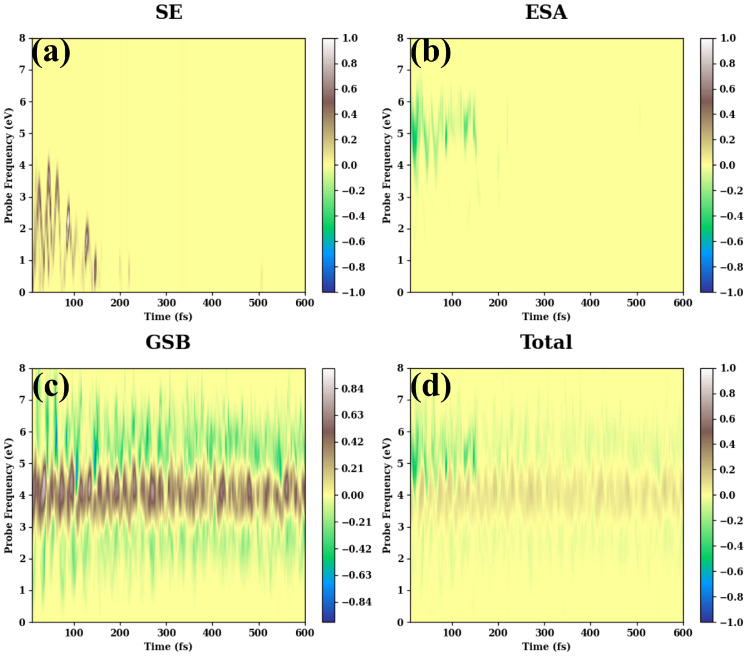
Normalized SE (**a**), ESA (**b**), GSB (**c**), and total (**d**) integral TA PP spectra of fulvene vs. pump-probe delay τ and the probe carrier frequency $\omega_{pr}$ evaluated up to 600 fs. The carrier frequency of the pump pulse, $\omega_{pu}=4.39$ eV, is in resonance with the $S_{1}$ state.

**Figure A3:**
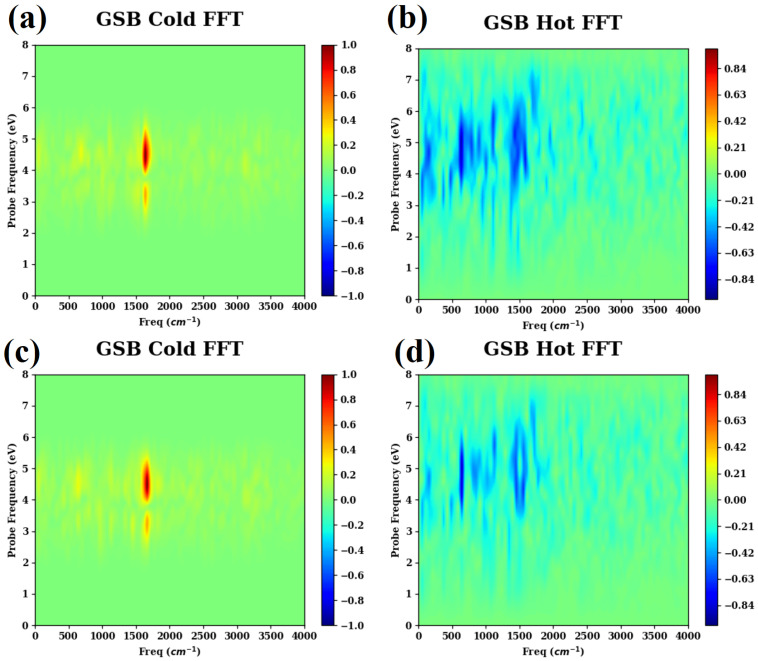
Fourier transforms of the normalized cold (**a**,**c**) and hot (**b**,**d**) GSB spectra of fulvene. The upper spectra are computed by integrating from 0 to 600 fs, while the lower spectra are evaluated by integrating from 10 to 600 fs.

**Figure A4:**
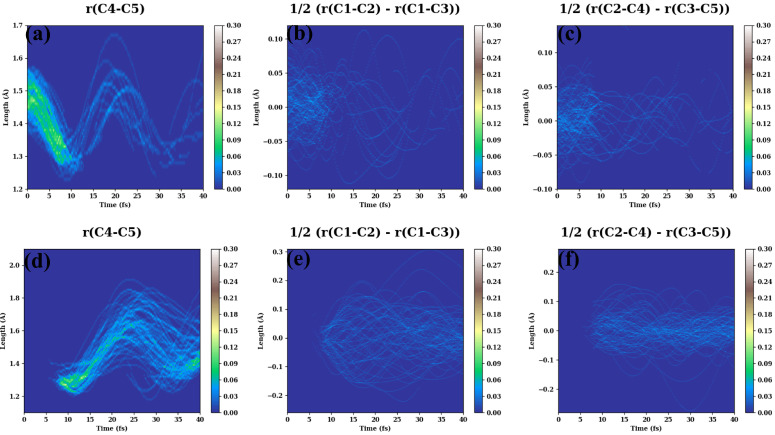
Normalized time-dependent distributions of C4–C5 stretch (**a**,**d**), C1–C2, and C1–C3 asymmetric stretches (**b**,**e**), and C2–C4 and C3–C5 asymmetric stretches (**c**,**f**). The upper panels are evaluated for the first excited state $S_{1}$, while the lower panels are evaluated for $S_{0}$.

**Figure A5:**
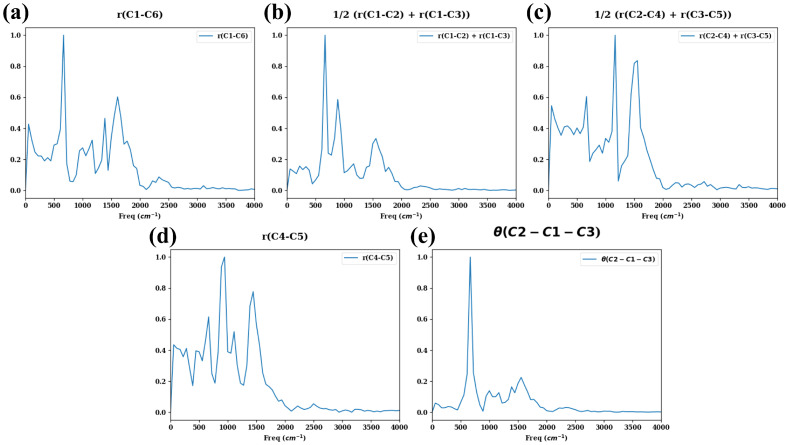
Normalized Fourier transform of (**a**) C1–C6, (**b**) C1–C2/C1–C3, (**c**) C2–C4/C3–C5, (**d**) C4–C5, and (**e**) C2–C1–C3 bending.

## Data Availability

The raw data supporting the conclusions of this article will be made available by the authors on request.

## Appendix A

CASSCF(6,6) level are shown in Figure A1.
The long-time TA PP signals are given in Figure A2.
The Fourier transforms of the cold and hot GSB signals are presented in Figure A3.
The evolutions of several additional internal coordinates are displayed in Figure A4.
Fourier transform of some critical nuclear motions is presented in Figure A5.
The excitation energies and TDMs between different electronic states are collected in Table A1.
The normal modes in the states $S_{0}$ and $S_{1}$ are summarized in Table A2 and Table A3, respectively.

**Table A1:** Energy gaps and TDMs between the selected states of the $S_{0}$ minimum at SA6-CASSCF(6,6)/6-31G(d) and the $S_{0}$ minimum optimized at SA2-CASSCF(6,6)/6-31G(d).

	Energy Gap (eV)	TDM (a.u.)
$S_{0}-S_{1}$	4.39	0.27
$S_{1}-S_{2}$	1.79	0.41
$S_{1}-S_{3}$	3.22	0.21
$S_{1}-S_{4}$	4.03	0.75
$S_{1}-S_{5}$	4.36	0.77

**Table A2:** Calculated vibrational frequencies of fulvene and their irreducible representations for the $S_{0}$ minimum at the SA2-CASSCF(6,6)/6-31G(d) level. The frequencies of these normal modes are suitable to analyze the cold GSB signals, instead of SE and ESA signals.

	$S_{0}$ Frequency cm^−1^ (Ranking)	Description
A1	1781.04 (24)	C=C, CH_2_ sciss.
	1637.5 (22)	C=C(r) CH_2_ sciss.,
	1563.95 (21)	C=C(r), CH_2_ sciss., C=C
	1483.03 (20)	CCH, C=C(r), C-C
	1194.15 (17)	CCH
	1048.43 (15)	C-C
	957.18 (13)	C-C
	706.37 (5)	CCC
A2	929.85 (12)	CH wag
	797.32 (8)	CCH_2_ tors, CH wag
	715.91 (6)	CCH_2_ tors, CH wag
	512.82 (3)	CCCC(r) def
B1	925.66 (11)	CH wag
	906.37 (10)	CH_2_ wag
	790.47 (7)	CH wag
	641.15 (4)	CCCC(r) def
	221.89 (1)	CCCC def
B2	1690.56 (23)	C=C(r)
	1461.33 (19)	C=C, CCH
	1368.43 (18)	C=C, CCH
	1187.5 (16)	CCH
	1037.95 (14)	CH_2_ rock
	856.37 (9)	CCC(r)
	369.08 (2)	CCC

**Table A3:** Calculated vibration frequencies of fulvene and their irreducible representations for $S_{1}$ minimum at the SA2-CASSCF(6,6)/6-31G(d) level.

	$S_{1}$ Frequency cm^−1^ (Ranking)	Description
A1	1705.61 (23)	C=C, CH_2_ sciss.
	1646.85 (22)	C=C(r) CH_2_ sciss.,
	1590.15 (21)	C=C(r), CH_2_ sciss., C=C
	1326.29 (18)	CCH, C=C(r), C-C
	1210.35 (17)	CCH
	1101.13 (16)	C-C
	1008.5 (14)	C-C
	679.71 (7)	CCC
A2	903.59 (11)	CH wag
	806.51 (10)	CCH_2_ tors, CH wag
	549.89 (6)	CCCC(r) def
B1	802.96 (9)	CH wag
	701.58 (8)	CH wag
	532.6 (5)	CCCC(r) def
	270.14 (3)	CCCC def
B2	1873.28 (24)	C=C(r)
	1485.52 (20)	C=C, CCH
	1388.54 (19)	C=C, CCH
	1088.25 (15)	CCH
	996.15 (13)	CH_2_ rock
	926.05 (12)	CCC(r)
	322.72 (4)	CCC
